# Supplementary material for: Are carbon emissions trading and green financial instruments synergistic? -Comprehensive quantitative research based on content analysis
Source: PLoS One. 2024 Mar 7;19(3):e0298601. doi: 10.1371/journal.pone.0298601 (PMC10919601; doi:10.1371/journal.pone.0298601)
Supplement: S1 Data — (ZIP) [file pone.0298601.s001.zip › Data/地级市Z_F工作报告.zip!/║╙─╧/╜╣╫≈╩╨/└┤╫╘╣1⁄2╓┌║┼í╛┬φ┐╦╩2╛▌═°í┐_2016.docx]

各位代表：

现在，我代表市人民政府，向大会报告工作，请予审议，并请各位政协委员和列席人士提出意见。

一、2015年及“十二五”工作回顾

2015年是“十二五”的收官之年。面对严峻复杂形势和经济

下行压力，在省委、省政府和市委的正确领导下，在市人大常委会、市政协的监督支持下，市政府紧紧依靠全市人民，全面落实党的十八大和十八届三中、四中、五中全会精神，深入贯彻习近平总书记系列重要讲话精神，坚持“凝心聚力、转型攻坚、争创一流、绿色发展”总要求和“一个战略、四项重点、十大建设”总思路，主动适应经济发展新常态，牢牢把握工作主动权，较好地完成了市十二届人大二次会议确定的主要目标任务。

据初步核算，全市生产总值1929亿元，增长9%；一般公共预算收入115亿元，增长9%；固定资产投资1883亿元，增长16%；社会消费品零售总额625亿元，增长12%；城乡居民人均可支配收入分别达到25895元、13682元，增长8%、9.3%，主要指标增速在全省位次前移，呈现稳中有进、稳中向好的发展态势。

一年来，我们主要做了以下工作：

（一）突出精准施策，稳增长、保态势取得明显成效。紧紧扭住发展第一要务，坚持解决当前突出矛盾和夯实长远发展基础相结合，多措并举，确保经济平稳健康运行。

强化政策支持。把实施“8+1行动计划”作为稳增长的总抓手，出台工业经济提速运行等九个方面的指导意见，制定促进经济稳定增长29条措施、促进房地产市场持续健康发展12条意见等，专题攻坚，扎实推进。

强化企业帮扶。建立市级领导分包十大产业工作机制，对规模以上工业企业全面调研摸底，因企施策，分类帮扶。成立依法处理工业后续问题工作小组，集中解决了一批企业遗留问题。

强化要素保障。组织银企对接会16次，设立1亿元应急转贷资金、3亿元产业发展基金，为73家企业提供“过桥”资金8.6亿元，为442家企业提供贷款担保21.7亿元。积极推进企业进入多层次资本市场，新增“新三板”挂牌企业10家。全年新增贷款91亿元，利用资本市场直接融资157亿元。保障土地供应，获批土地1.3万亩。规模以上工业增加值1105亿元，增长10.2%。

（二）推进十大建设，发展后劲不断增强。以十大建设为重点，强力实施项目带动，206个中央预算内投资项目全部开工，35个省重点项目、204个市重点项目分别完成投资185亿元、558亿元，超额完成年度计划。

城市水系方面，13个重点项目加快推进，大沙河从出山口至吴泽湖段40公里全线贯通，新河源头河道治理和堤顶道路完工，影视湖公园、白鹭湿地公园建成，瓮涧河丰收路至龙源路段景观改造工程完工，城区形成水面8000亩、湿地5000亩，我市成为国家水生态文明城市建设试点。

铁路方面，郑焦铁路通车运营，火车站南广场开工建设，焦作直达北京高铁正式开通，太焦客运专线、新焦济洛城际铁路前期工作加快推进，开启了我市以高铁为代表的大交通时代。

公路方面，焦作至荥阳黄河公路大桥及连接线工程全面启动，武云高速主体完工，南太行绕城高速前期工作基本完成。

能源方面，中原天然气综合利用产业基地建设加快推进，神华焦作电厂、华润龙源电厂建成投运，中电投丹河电厂项目开工。

信息化方面，“无线城市”“智慧焦作”建设14个项目完成投资14.6亿元，“全光网焦作”提前实现。

示范区和产业集聚区、生态、教育、文化、开放型经济等方面，一批重大项目加快实施。加强项目谋划，发布推介总投资1038亿元的十大战略项目。项目建设的顺利推进，既为稳增长、保态势提供了有力支撑，也为创优势、促转型奠定了坚实基础。

（三）加快转型升级，推动经济质量和环境质量双提升。面对经济下行和环境保护双重压力，坚持把转型升级放在突出位置，标本兼治，调转结合，不断提高发展的质量和效益。

工业结构加快调整。围绕十大产业，重点实施100个转型升级项目，完成投资182亿元，蒙牛纯甄生产线、光源电力LED产业园等项目建成投产。围绕传统产业改造升级，大力实施223个技改项目，完成投资236亿元，中铝中州产能优化、佰利联氯化法钛白粉等项目竣工投产。围绕培育新兴产业，加快多氟多锂电池、健康元医药中间体等项目建设，制订实施新能源汽车产业发展规划。围绕两化深度融合，实施示范项目25个。开展质量提升工程，新增中国驰名商标2件。十大产业增加值增长13.4%，对工业贡献率达82%。

现代服务业亮点纷呈。电子商务蓬勃发展，腾云电子商务产业园一期建成，浪潮云计算中心、阿里巴巴产业带等75个项目落户。博爱成为首批全国电子商务进农村综合示范县，孟州成为全省首个农村淘宝试点县。全市电商企业达1645家，交易额545亿元。创建省级电子商务示范基地1家、示范企业6家，建成电商园区8个。现代物流业快速发展，河南德众保税物流中心封关运营，焦作（中站）物流金融港等8个重点园区建设加快推进，AAAA级物流企业实现零的突破，新增省级物流示范园区2家，快递业务增长70%。金融业规模不断壮大，焦作中旅银行正式揭牌，中原银行焦作分行即将开业，实现与上海证券交易所、上海期货交易所的合作对接。文化旅游业转型升级加快，谋划打造“中国养生地·世界太极城”，文化旅游十大园区完成投资19.8亿元，青天河休闲度假中心、嘉应大观园等项目加快推进，云台山获得中国质量奖提名奖。成功创建国家级旅游服务标准化示范市，年接待游客3741万人次，旅游综合收入302亿元，分别增长13.4%、15.4%。促进房地产市场健康发展，发放公积金贷款19亿元，完成商品房销售1.2万套。

农业发展方式持续转变。新建高标准粮田28万亩，粮食总产达209万吨，实现“十二连增”，被命名为中国优质小麦之都。实施农业产业化重点项目48个，农业产业化集群销售收入达602亿元。百千万农民创业创新行动全面启动。新增农民合作社449家，覆盖率超过50%。农村土地流转率达53.3%，高于全省17个百分点。新增家庭农场156家，建设都市生态农业园区16个。选育农林作物新品种1个、新品系9个。主要粮食综合机械化水平保持在96%以上，居全省前列。

载体建设成效明显。焦作高新区升级为国家高新区。示范区成功创建省级服务外包示范园区。产业集聚区固定资产投资1300亿元，增长22.2%。沁阳、武陟产业集聚区获批省级经开区，孟州、温县等4个产业集聚区进入全省集聚区集群发展30强，数量并列全省第一。服务业“两区”建设有序推进，新河商务中心区建设全面启动，万达广场项目落地开工。

创新支撑能力不断增强。实施省重大科技专项5个，获省科技进步奖15项。焦作科技大市场建成投用，全省商行系统首家科技支行挂牌运营，与大连理工大学合作建立装备制造技术研究中心。授权专利1420件，引进高层次紧缺人才961人。新增国家级高新技术企业21家。高技术产业增加值增长22.5%。

生态文明建设扎实推进。深入开展“2015蓝天雷霆”行动，强力推进十大专项治理，完成工业企业大气污染深度治理329家，整治建筑工地扬尘155家，拆改燃煤锅炉510台，关停煤场773家，淘汰黄标车9179辆。圆满完成“9·3”阅兵、上合组织政府首脑理事会议环境保障任务。大力推进清水工程，深化海河等重点流域污染治理，市第二污水处理厂扩建完工，第一污水处理厂迁建加快推进。实施乡村清洁工程，推进农村环境连片整治，开展生态创建项目56个。创建国家森林城市成效显著，北部山区生态综合治理深入推进，完成森林培育16.9万亩，森林覆盖率达30.9%。持续推进节能减排，预计单位生产总值能耗下降0.88%，主要污染物减排完成省定控制目标。

（四）深化改革开放，动力活力持续释放。把改革开放作为加快转型发展的关键举措，坚定不移深入推进。

各项改革务实推进。大力简政放权，扎实开展全省行政审批标准化试点市创建，各类审批事项由170项压减到136项，市直部门权力清单发布全面完成，乡、村便民服务体系更加完善。完成政府机构改革。实行“三证合一、一照一码”，新增市场主体2.7万户，增长29.4%。加快投融资机制创新，推出34个总投资424亿元的PPP项目，市投资集团50亿元债券获批发行。在全省率先建立“一委、一办、一中心”的公共资源交易组织架构，完成交易项目955个，增收节支4亿元。深入开展综合治税，增加税收7.9亿元。我市成为国家城市公立医院综合改革试点市，全科医生执业方式和服务模式改革深入推进。党政机关公务用车制度改革、农村土地承包经营权确权登记等改革基本完成，交通运输执法和运管体制改革等全面完成。

开放步伐持续加快。强力实施“333”招商行动，依托本地商会、驻外商会、行业协会，重点围绕珠三角、长三角、京津冀，突出文化旅游、科技、电子商务、先进制造等专题，大力开放招商，引进市外境内项目862个，总投资1568亿元。焦作海关启动丝绸之路经济带区域海关通关一体化模式，形成口岸大通关便捷通道。建成首个国家级出口四大怀药质量安全示范区，新增省级出口基地2家。进出口总额20.4亿美元，居全省第2位。

（五）统筹城乡发展，新型城镇化有序推进。充分发挥新型城镇化“一发动全身”的综合带动作用，加快城乡一体化发展。

体制机制创新力度加大。深化户籍制度改革，完善就业、教育、医疗等配套政策，农业转移人口市民化步伐加快。与国开行、省交行合作设立新型城镇化发展基金。孟州成为省新型城镇化综合试点。4个镇入围全省第一批重点示范镇。

城市基础设施不断改善。实施53个总投资97.2亿元的城建项目。龙源电厂配套供热管网建设完成，主干管网覆盖率从20%提高到78%，集中供热普及率从20%提高到40%，实现历史性提升。新改建供水管网29公里。大力实施“气化焦作”工程，新改建燃气管网74公里。编制完善中站、马村污水管网规划。龙源路东段等建成通车，丰收路、中原路等改造完成。购置新能源公交车103台。深入开展市容环境专项整治行动，城市管理水平进一步提升。

美丽乡村建设加快推进。新改建农村公路527公里，改造农村危房5295户，建设各类饮水工程143处，解决农村17.4万人饮水安全问题。建成农村人居环境达标村587个、示范村312个。完成158个扶贫开发项目，实现1.46万人脱贫。

（六）倾力改善民生，社会大局保持和谐稳定。民生领域财政支出达161亿元，增长10.9%，占一般公共预算支出的74.4%，省市十项重点民生工程全面完成。

就业创业稳步推进。大力实施全民技能振兴工程，举办就业创业春风行动等活动，推动大众创业、万众创新，发放创业担保贷款5亿元，城镇新增就业9万人，农村劳动力转移就业3.7万人，我市成为全国第二批百城技能振兴专项活动试点市。

社会保障更加有力。深入开展对城乡低保、困难群体的临时救助和商业保险医疗救助，继续推行城镇医保按比例报销上不封顶制度，1426名大病患者直接受益；职工大额补充医疗保险实行即时结算，新农合大病保险全面实施。城乡居民养老保险参保率达99.8%，居全省第2位。企业退休人员基本养老金人均每月达1950元。基本建成保障性住房7442套，新开工5707套。住房公积金覆盖面达86.7%。发放城乡低保金、农村五保金2.5亿元。市福利院搬迁和老年公寓楼建设取得实质性进展，5个县级社会福利中心、5个残疾人就业培训基地建成投用。实施残疾人、65岁以上老年人免费乘公交政策，惠及近8万人。

社会事业加快发展。4个县区通过国家义务教育发展基本均衡县区验收，丰泽园小学、北清高中等一批中小学建成投用，组建73个义务教育集团，完成100个农村中小学标准化项目，成立市职业教育集团。医疗联合体建设加快推进，市二院新区医院、妇幼保健院综合病房楼等项目开工建设。在107个村（社区）开展基层综合性文化中心建设试点，实施文化惠民演出386场。成功举办“一赛一节”和省第十二届老年人体育健身大会，完成省全民健身示范市创建；组织举办“共享太极·共享健康”世界百万太极拳爱好者共同演练活动，创造新的吉尼斯世界纪录。

平安焦作建设取得新成效。加强社会治安综合治理，保持对各类违法犯罪的高压态势。开展安全隐患集中排查整治，全面强化安全生产，积极推进食品药品监管体制改革，严厉打击和处置非法集资，依法依规解决信访问题，提升应急管理水平，人民群众安全感和满意度稳步提高。

一年来，我们深入开展“三严三实”专题教育和“三查三保”活动，持续加强政府自身建设，专项整治懒政怠政为官不为，改进工作作风，规范行政行为。主动接受人大及其常委会监督，积极支持政协参政议政，全年办理人大代表建议113件、政协委员提案382件，质量和效果不断提高。大力推进依法行政，健全行政机关负责人出庭应诉等制度。加强政府系统廉政建设，强化对权力运行的制约和监督。“三公”经费下降23%。同时，双拥工作取得新成效，军民融合发展质量和国防动员保障能力进一步提升。外事侨务、台湾事务、民族宗教、统计调查、审计、档案、史志、气象、地震、人防、红十字等工作，都取得了新成绩。

各位代表！

2015年各项工作目标的实现，标志着我市“十二五”主要任务胜利完成。这五年，我们走过的历程极不平凡，遇到的困难比预料的更大，取得的成绩比预想的更好。

这五年，是综合实力再上台阶的五年。预计2015年，生产总值比2010年提高63.5%，年均增长10.4%。人均地区生产总值54457元，是全省平均水平的1.4倍。一般公共预算收入是2010年的1.8倍。规模以上工业增加值是2010年的1.9倍。

这五年，是转型示范成效显著的五年。产业集聚区经济总量占全市60%以上。高新技术产业增加值比重达40%。第三产业增加值占比由26.2%提高到32.4%。示范区成功创建国家新型工业化产业示范基地。实现全国科技进步先进市六连冠，连续四次获得中国旅游百强城市称号。

这五年，是城乡面貌明显改观的五年。城镇化率由47.1%提高到54.5%，高于全省平均水平8个百分点。中心城市建成区由95平方公里扩大到116平方公里。新建龙翔矿山、黎明脚步等公园，建成城市游园26个，城市绿化覆盖率达40%，人均公共绿地达11.8平方米。成为全省首批生态文明先行示范市。

这五年，是改革开放深入推进的五年。行政审批、公共资源交易等改革走在全国全省前列，财政“四权分离”获中国地方政府创新奖。实现与中国兵装、奇瑞、港中旅、中关村等战略合作，交通、光大、广发等一批金融机构落户，焦作海关开关运营。

这五年，是人民生活持续改善的五年。累计民生支出657亿元。2015年城乡居民人均可支配收入分别是2010年的1.6倍和1.8倍。成为全国推进义务教育均衡发展工作先进市，覆盖城乡的社会保障体系和多层次住房保障体系加快完善，人民群众得到更多实惠。

特别是，我们干成了很多打基础利长远的事情，干成了一些多年来一直想干的事情，取得了一系列重大突破。经济转型示范市上升为省级战略，郑焦城际铁路、桃花峪黄河大桥建成通车，交通区位优势更加凸显；南水北调和引黄入焦总干渠全线贯通，“六纵四横”内部大循环、外部大联通的城市水系功能正在显现；太极体育中心、职教园区一期、科技馆、图书馆等投入使用，城市公共服务功能不断提升。经过五年不懈努力，我们的发展思路更加清晰，发展方式更加科学，发展基础更加坚实，经济转型示范市和美丽焦作建设站上新的起点，展现出更加美好的前景！

各位代表！

“十二五”成绩的取得，是省委、省政府和市委正确领导的结果，是市人大、市政协、焦作军分区大力支持的结果，是全市广大干部群众凝心聚力、共同奋斗的结果。在此，我代表市人民政府，向辛勤工作在各条战线的全市人民，向人大代表、政协委员，向各民主党派、工商联和无党派人士、各人民团体，向驻焦部队、武警官兵和公安干警，向离退休老领导、老同志，向所有关心支持焦作发展的社会各界人士，表示衷心的感谢！

在总结成绩的同时，我们也清醒地认识到，我市仍处于爬坡过坎、转型攻坚的关键时期，发展中还存在不少困难和问题。一是经济下行和环境保护压力持续加大的局面短期难以缓解，保增长和保生态任务艰巨。二是转型升级的要求更为迫切，供需错配的矛盾更为突出，一些企业生产经营困难加剧，改造提升传统产业、加快培育新的增长动力刻不容缓。三是发展环境不优、体制机制不活、市场体系不健全等问题还不同程度存在，在区域竞争中面临更大挑战。四是基础设施建设和民生事业仍比较薄弱，一些关系长远发展和群众切身利益的问题尚未有效解决。五是政府职能转变、简政放权还不到位，一些工作人员服务意识、能力素质、工作作风与发展要求和人民期盼还不适应，不作为、不愿为、不会为依然存在。一些腐败现象仍时有发生。对此，我们一定高度重视、勇于担当、认真解决，决不辜负人民期望！

二、“十三五”发展总体思路和目标任务

“十三五”时期是全面建成小康社会的决胜阶段。这一时期，外部环境依然复杂多变，但我市仍处于可以大有作为的重要战略机遇期。一是国际产业分工格局加速调整，“互联网+”等新一轮科技革命和产业变革正在孕育兴起，产业转移仍将持续，有利于我们实施创新驱动，引进培育新的产业支撑力量。二是国家实施“一带一路”战略，提出把中原地区培育成带动发展的新空间，出台促进中部崛起新十年规划，继续支持资源枯竭城市转型和老工业基地建设，有利于我们进一步扩大开放，形成政策叠加优势。三是我省深入实施三大国家战略规划，构筑“一极三圈八轴带”发展新格局，有利于我们对接融合，提升焦作在区域发展中的地位和作用。四是我市广大干部群众思转型谋发展、做示范求跨越的积极性日益高涨，同心同德、务实重干的氛围更加浓厚，有利于我们更好抢抓机遇，以新作为开创发展新局面。

“十三五”时期经济社会发展的指导思想是：全面贯彻党的十八大和十八届三中、四中、五中全会精神，深入贯彻习近平总书记系列重要讲话精神，认真贯彻省委全会和省委经济工作会议精神，按照“五位一体”总体布局和“四个全面”战略布局，牢固树立和坚决落实五大发展理念，坚持和完善“凝心聚力、转型攻坚、争创一流、绿色发展”总要求和“一个战略、四项重点、十大建设”总思路，适应新常态，抢抓新机遇，促进新转型，在全面建成小康社会进程中努力走在全省前列，为实现经济转型示范市和美丽焦作建设奠定坚实基础。

战略定位和发展目标是：加快建设中原经济区经济转型示范市，努力把焦作打造成为更具活力、更具吸引力、更具竞争力的新型工业城、区域中心城、知名旅游城、生态宜居城、太极文化城。力争主要经济指标年均增速高于全省平均水平，生产总值年均增速高于全省平均水平1个百分点以上，力争达到9%左右，一般公共预算收入年均增长8%左右，争取经济总量进入全省第一方阵，努力迈入前五行列，提前实现地区生产总值、城乡居民人均可支配收入比2010年翻番目标。

各位代表！

《焦作市国民经济和社会发展第十三个五年规划纲要（草案）》及说明已经印发大会，请予审议。

我们坚信，有省委、省政府和市委的坚强领导，有全市人民的齐心协力、奋勇拼搏，再大的困难也阻挡不了我们前进的步伐，我市“十三五”宏伟蓝图一定能够实现！

三、2016年发展目标和重点工作

今年是全面建成小康社会决胜阶段的开局之年，也是推进结构性改革的攻坚之年。我市面临的经济下行压力加大，自身的结构性矛盾更为突出，但变中有机、逆中有势、难中有顺。我们必须坚定信心，保持定力，以滚石上山的拼劲、抓铁有痕的韧劲，全面贯彻中央加强供给侧结构性改革的决策部署，认真落实省委持续抓好“四个一”“五个点”的要求，统筹稳增长、促改革、调结构、强基础、惠民生、防风险，突出去产能、去库存、去杠杆、降成本、补短板，着力提高供给体系质量和效率，改造提升传统比较优势，加快培育新的发展动能，确保“十三五”实现良好开局。

今年主要预期目标是：生产总值增长8.5%左右，一般公共预算收入增长7.5%左右，固定资产投资增长16%，社会消费品零售总额增长12%，进出口总值增长5%，城镇新增就业6万人，城乡居民收入增长与经济增长基本同步，节能减排、环保等完成省定任务，发展质量和效益明显提高，结构性改革取得实质性进展。

实现上述目标任务，必须全面贯彻落实市委总要求和总思路，突出“一个战略”，做到“四个坚持”。“一个战略”，就是继续以经济转型示范市这一战略统领各项工作，加快建设美丽焦作。“四个坚持”，就是坚持凝心聚力这一前提，把思想和行动统一到中央和省委、市委的决策部署上来，聚焦发展第一要务，汇聚起全市人民干事创业的强大正能量。坚持转型攻坚这一关键，将经济发展与环境保护的“双压力”由矛盾对立关系统一到转型升级上来，努力实现“双提升”，做到换挡不减势、量增质更优。坚持争创一流这一目标，大力弘扬敢打善拼、不甘人后的拼抢精神，自我加压、奋力赶超，加快实现升位晋级。坚持绿色发展这一目的，走绿色、循环、低碳发展之路，让人民群众享受到舒适舒心、宜居宜业的环境，拥有更多的获得感和幸福感。

今年，着力抓好八个方面工作：

（一）着力深化改革开放。推进改革创新，持续增强发展内生动能；坚持开放带动，不断提升内外联动发展能力。

全面深化改革，破解发展难题。主动承接上级各项改革举措，扎实稳步推进，确保落地生根。更加注重精准发力，聚焦解决具体问题，聚焦增添发展动力，聚焦构建发展新体制，在经济社会发展方面争取更多试点，力求率先突破。进一步简政放权，深化行政审批制度改革，实行权力清单动态管理，探索建立行政审批事中事后监管机制，清理规范中介服务，实现“三证合一、一照一码”两日办结，积极申建全国行政审批标准化试点市。实现公共资源交易管办分离，建立市县一体的交易、服务和监督平台，加快区域性交易平台建设。推进不动产登记制度改革，保障合法产权。深化财税管理体制改革，大力培植财源、促进发展；推进预算公开，加快中期财政规划管理改革；加强社会综合治税，推动国税地税合作。加快投融资改革，发挥财政资金杠杆作用，激活撬动社会资本，抢抓中央适度增加地方政府发债规模机遇，积极争取支持；扩大PPP应用领域，建立PPP专项基金、中小企业发展扶持基金等，用好新型城镇化、新型产业化、旅游产业基金，健全政府引导基金体系。深化文化体制改革，组建报业传媒集团和广电传媒集团。加快社会信用体系建设，开通运行信用焦作网站。同时，加快推进教育、卫生、机关事业单位养老保险、县以下机关公务员职务职级并行制度等各项改革。

突出链式招商，打造产业集群。把引入龙头企业作为衡量招商成效的核心标准，以装备制造、新能源汽车、文化旅游、电子商务、特色农业等优势产业为重点，紧盯国内外500强、央企和行业龙头，靠大联强，延链补链，发挥现有企业优势，加快引进与我市优势产业相配套的重点项目，努力打造千亿先进制造业集群、高成长性服务业集群、高附加值现代农业产业集群。

创新招商模式，提高招商实效。坚持集群招商、驻地招商、定向招商，更加注重发挥企业、商会、协会作用以商招商，注重组建专题招商团队精准招商，注重支持各类产业园区整体招商，注重鼓励现有企业增资扩股零地招商。积极参加河南投洽会、厦洽会、广交会，办好第十一届豫商大会等招商活动。落实招商引资重大项目四评工作机制，狠抓项目落地。

对接国家战略，拓展外向型经济空间。抢抓“一带一路”重大机遇，融入全省开放大格局，充分借力郑州航空港实验区枢纽和口岸体系，引进出口型临空产业项目，加快产业对接和服务配套。加强省级出口基地和服务外包示范园区建设，创建出口质量安全示范区，争取进口肉类指定口岸查验区落地，疏通连接郑州空港、郑欧班列陆港的运输体系，发展连通东部海港、西部陆港的口岸业务。依托郑州跨境电子商务综合试验区申建，重点推进德众跨境电商园、韩国跨境电商平台等项目落地，培育外贸竞争新优势。

优化营商环境，提升服务企业水平。把优化环境作为开放招商的生命线，研究吃透、用足用好上级政策，推进政策创新，营造公平透明可预期的政策环境；强化效能建设，提高办事效率，营造优质高效的服务环境；规范行政执法行为，整顿市场秩序，保护投资者合法权益，营造公正严明的法治环境。全面推行外来投资项目联审联批制和无偿代理制，实行项目落地一把手负责制；帮助企业降低成本，强化资金、土地、水电气等要素保障，确保项目招得来、留得住、做得大。在服务企业上，无论是本土企业还是外来企业，无论是大企业还是中小微企业，都一视同仁，做到“无事不扰、有求必应”，决不能吃拿卡要，决不能私立名目、乱收费用，建立“一站式”服务平台，确保企业诉求件件有着落、事事有回复，真心实意、竭尽所能助力企业腾飞发展，让服务企业成为焦作的“金字招牌”，让发展环境成为焦作的“亮丽名片”。

（二）着力厚植发展优势。把厚植优势作为引领发展的着眼点和立足点，推动单一优势、局部优势向综合优势转变。

突出项目带动。切实抓好产业项目，积极谋划推动一批市场前景好、技术含量高、带动能力强的重大产业项目落地建设，提升产业竞争力，增添发展动能，夯实发展基础。继续实施好城市水系、铁路、公路、生态等十大建设，加大省市重点项目谋划推进力度，强化发展支撑。加快现代综合交通体系建设，重点推进太焦客运专线、新焦济洛城际铁路、焦作至荥阳黄河公路大桥及连接线、火车站南广场等项目，确保武云高速建成通车，力争南太行绕城高速开工，加快孟州至武陟沿黄快速通道前期工作，努力构建市外联通大循环、市域互通中循环、市区畅通小循环的大交通格局，让道路更畅通、让出行更方便。更加注重投资精准性和有效性，政府重点谋划推进打基础、管长远、惠民生的大项目，支持投融资平台改革创新、转型发展、做大做强；尊重企业投资主体地位，市场能办的多放给市场，推动项目建设更有效率、更可持续。

强化载体支撑。加快示范区发展，突出投资拉动，培育新兴业态，推进城市功能区综合连片开发，强化产业和功能复合，努力走进全省先进行列。推动产业集聚区提质发展，突出集群、创新、智慧、绿色发展，加快由规模扩张向质、量并重转变，围绕主导产业延伸产业链条，推进区镇管理套合，提升吸引力竞争力带动力，争取更多产业集聚区进入全省十强、十快、十先进。开展服务业“两区”示范创建活动，力争在建项目超过30个，千万元以上项目投资突破30亿元，基础设施投资完成5亿元。

实施创新驱动。大力推进开放式创新，积极创建国家自主创新示范区，更好发挥科技创新引领支撑作用。突出创新重点，围绕优势产业，集中创新资源，谋划实施一批重大科技专项，争取在新能源汽车、智能制造等领域实现突破。建好创新平台，加快大学科技园建设步伐，引导和激励科研机构、高校、企业积极建设工程技术研究中心、院士工作站、重点实验室等研发平台，开展产学研合作和协同创新，提升企业创新能力和水平。完善创新机制，健全科技企业融资担保、贷款贴息、风险补偿等制度，加快科技成果转化和产业化进程。发挥“双创”汇众智、聚众力的乘数效应，建立健全创业创新优惠政策和服务体系，构建众创、众包、众扶、众筹支撑平台，孵化一批创新型小微企业。研究制定人才柔性引进政策，注重以重大攻关项目、战略发展项目带人才，以招标等方式引进有特定技术的人才或团队创新创业。

壮大县域经济。把县域作为全面建成小康社会的战略重点，努力创造条件激发县域发展活力，调整优化市与县市区财税共赢体制，支持各县市区发挥优势壮大主导产业，错位联动发展，再造县域经济发展新优势，为全市跨越发展提供重大支撑。

（三）着力推动工业转型升级。坚持工业强市，聚焦供给侧结构性改革，优化存量与培育增量并举，以智能制造为主攻方向，推进两化深度融合，努力实现产业水平整体跃升。

打造六大产业集群。以发挥特色优势为重点打造装备制造产业集群，做大做强矿山装备、工程机械等产业，努力在通用航空、智能装备领域实现突破，重点推进正旭航空航天铝合金构件、焦矿重型成套装备等项目建设。以发展新能源汽车为重点打造汽车及零部件产业集群，推动新能源汽车电池、电机、电控三大技术研发和生产，壮大汽车关键部件、专用车等产业，形成配套优势，重点推进多氟多新能源汽车动力总成、风神轮胎轿车子午胎等项目建设。以延链补链为重点打造现代化工产业集群，以发展循环化工、精细化工为方向，加快化工产业绿色化改造，突出降本增效，重点推进佰利联氯化法钛白粉扩建、晋煤天庆煤制天然气等项目建设。以塑造品牌为重点打造食品产业集群，巩固提升乳制品、面制品、肉制品等传统产业，积极培育功能饮料、休闲食品、保健食品等新兴产业，重点推进蒙牛液态奶扩建、明仁保健品等项目建设。以高端化、规模化为重点打造生物医药产业集群，发展头孢类抗生素、核黄素等特色产业链，立足四大怀药原产地优势，提高怀药产品产业化程度，打造中国怀药之都，重点推进丽珠生物医药中间体、保和堂中药饮片等项目建设。以技术突破为重点打造新材料产业集群，大力提升研发水平，做精做强超硬材料、合金材料等特色产业链，重点推进大地合金航空精密棒材、金桥白鸽磨料磨具等项目建设。

培育发展新兴产业。加快发展节能环保产业，重点开发高效节能产品、余热余压利用设备，建设国家资源综合利用双百工程示范基地。加快发展电子信息产业，大力引进智能终端、新型显示设备生产配套企业，积极发展电子元器件、通信电缆、导航设备等产品。加快发展新能源产业，壮大天然气、煤层气发展规模，打造风光电组件及关键零部件制造产业链。

改造提升传统产业。推动铝工业、煤炭、轻工纺织等传统产业转型发展，支持企业大力开拓市场，调整产品结构，加快技术改造，强化成本管控，重点推进中州铝业化学品氧化铝扩建、奋安铝业高档铝型材等项目建设。妥善定性与处置“僵尸企业”，多兼并重组、少破产清算，对产品有市场、有效益但暂时遇到困难的企业，给予大力支持，帮助其渡过难关。

实施工业转型升级800行动计划。支持100户重点企业发展，加强100个重点转型升级项目建设，培育100家两化融合示范企业，扶持100家小微企业快速成长，帮助100家困难企业解决实际问题，推进100家企业股份制改造，开展100家中小企业投融资服务，提升100位企业家综合能力素质，加快工业提质增效发展步伐。

（四）着力促进现代服务业提速发展。推动生产性服务业向专业化和价值链高端延伸、生活性服务业向精细化和高品质转变，把服务业发展的巨大潜力转化为现实生产力。

推进电子商务创新发展。加快电子商务中心区和电商产业园区发展，重点推进腾云电商产业园二期、阿里巴巴产业带、新浪区域运营中心等项目建设。积极创建省级、国家级电子商务示范园区。开展电子商务进农村、进企业、进社区活动。全市全年电子商务交易额力争达到600亿元以上。

推进金融业突破发展。大力发展多元金融，深化与金融机构有效合作，鼓励金融机构与优势产业融合发展，创新产品、创优服务。推进农村信用社改制农商行，推动村镇银行县域全覆盖。发展消费金融、绿色金融和金融租赁等新兴业态。深化与上海期货交易所合作，加快筹建焦作大宗商品交易所。推动企业进入多层次资本市场，提高直接融资比重。优化金融生态环境，提升金融服务实体经济效率。

推进文化旅游业跨越发展。围绕建设知名旅游城、太极文化城，努力打造集生态观光、休闲度假、健康养生、山地运动为一体的南太行生态旅游集聚带；打造集历史文化游、生态观光游、民俗民情游为一体的黄河文化旅游产业带；加快南水北调城区段两侧控规区规划设计和改造开发，打造生态休闲带；推进大沙河生态湿地建设，打造湿地景观带。规划建设特色餐饮、文化、娱乐休闲街区，形成城区旅游新亮点。重点推进云台山文化旅游园区、黄河（嘉应观）文化旅游区、青天河（月山寺）园区休闲度假中心、妙乐寺文化旅游园区等项目建设，启动实施云台山文化旅游展演项目。筹建太极拳文化产业平台，大力发展太极拳产业。做优绞胎瓷、山阳刺绣等特色文化旅游产品。发展智慧旅游，完善国际旅游目的地公共服务体系。支持博爱、修武创建国家全域旅游示范县。

推进现代商贸物流业融合发展。加快物流园区智能化、信息化和标准化改造，规划建设与电商配套的快递物流园区，重点推进焦作（中站）物流金融港、河南（武陟）国家干线铁路公路物流港、德众大罗塘国际农产品集散中心等项目建设。抓好物流企业培育，力争新增5家AA级以上企业。推动快递下乡，健全城乡物流配送体系。推动连锁经营、物流配送和电子商务协同发展，扩大家政、养老等领域消费，积极推进万达广场等城市综合体项目建设。

推进房地产业健康发展。鼓励住房消费，研究制定更具针对性的政策措施，加快消化房地产库存，扩大公积金覆盖面，鼓励农民进城购房，发展住房租赁市场，畅通商品房供给保障性住房通道。

（五）着力发展现代农业。加快转变农业发展方式，以供给侧结构性改革强农惠农富农，推动农村一二三产融合发展，实现稳粮增收、提质增效。

提高粮食综合生产能力。落实藏粮于地、藏粮于技战略，加强农田水利设施建设，发展节水灌溉6万亩，加快黄河防洪工程及下游河道治理工程建设，提升机械化水平，强化气象为农服务，加快高标准粮田建设，确保粮食稳产高产。

调整农业结构。推动粮经饲统筹、种养加一体融合发展，促进互联网与现代农业深度融合，发展都市生态农业、智慧农业，规划建设一批精准设施园区、高效农业示范园区，加快形成结构合理、品种和质量契合消费者需要的农产品有效供给。

壮大农业产业化集群。重点支持6个国家级、39个省级龙头企业发展壮大，规划建设一批以农产品初加工、特色产品加工为重点的特色园区，发展一批主食产业化、畜牧产业化、种子产业化特色产业集群。继续开展百千万农民创业创新行动，建成40个创效型产业村、发展440家创新型企业、培育4400名创业型农民。以发展农民合作社和家庭农场为重点，引导土地承包经营权有序流转，大力推进农业适度规模经营。

创建农产品品牌。加强农畜产品质量安全全过程监管，大力推动农业标准化生产，积极开展“三品一标”认证，加快建立农畜产品质量安全追溯信息平台。开发一批市场认可、消费者信赖的优质农畜产品，打造一批竞争力强、影响面广的知名品牌。

深化农村改革。落实农业三项补贴改革，做好农村土地承包经营权、宅基地使用权确权登记颁证后续工作，建立农村产权流转交易市场，深化农村金融改革，大力推广农业保险，加快推进温县省级现代农业综合配套改革试验区建设。

（六）着力加快新型城镇化进程。坚持产城相辅、人城相融、城市规模与资源环境相适应，以产兴城、依城促产，推动城镇化提质提速发展。

优化城镇空间布局。坚持核心提升、廊带集聚、多点支撑，推进形成“一核两廊四带多点”的空间发展布局。进一步推动郑焦融合发展，优化郑焦高铁、高速、黄河大桥沿线产业布局。加快推进焦修博武一体化。全面开展城市设计，推进多规合一，强化规划刚性，重点抓好旧城更新、轨道交通、城市停车场等规划编制实施，打造疏密有致、开合有度、生态宜居的空间格局和城市风貌。

增强城市承载能力。坚持中心城市带动，完善城市基础设施，有效改善市民生产生活条件。实施中原路、民主路等城市道路新建维修工程，谋划打通一批断头路，新购置308台绿色环保公交车，大力治理城市交通拥堵。开工建设府城水厂一期和博爱输水线路项目，实施群英河、瓮涧河整治工程。加强城市地下空间开发利用，推动地下综合管廊建设，统筹电力、通信、广播电视、给排水等市政管线建设和管理。新建一批燃气管网和供水管网，建设龙源电厂供热管网二期、示范区供热管网一期等项目。开工建设西部产业集聚区污水处理厂及中站、马村污水管网。加大城中村、棚户区和危房改造力度，新开工保障性住房1325套，推进棚户区改造货币化安置，加快推进老旧小区综合整治和集中连片改造。创新城市管理体制机制，加大环卫公共设施投入，加强市容环境整治。积极推进餐厨废弃物处置、垃圾焚烧发电等项目筹备工作，不断提高城市精细化、规范化、数字化、法治化管理水平。

推进城乡一体发展。完善县城和中心镇产业功能、服务功能、居住功能，增强承接中心城市辐射和带动乡村发展的能力。加强农村基础设施建设和社会事业发展，持续改善农村生产生活条件。推动特色小镇创新发展，打造一批基础设施完备、生态环境优美、富有地方特色的美丽乡村。实施一批省道改建项目，新改建农村公路400公里，确保郑常线等改建工程完工。

加快农村转移人口市民化。突出“一基本两牵动三保障”，加快户籍制度改革和居住证制度落地，切实维护进城落户农民的土地承包权、宅基地使用权、集体收益分配权，加大农民转移就业培训力度，不断提升农民工转化为市民的能力，努力缩小户籍人口城镇化率和常住人口城镇化率差距。

（七）着力加强生态环境保护。环境污染是发展之患、民心之痛，必须铁腕治理。坚持源头严防、过程严控、后果严惩，加强生态体系建设，大力推进绿色发展。

实施蓝天工程。继续深入开展十大专项治理，坚持治理一批、搬迁一批、转产一批、关闭一批，完成35家城区工业企业综合整治任务，对12家企业燃煤机组进行超低排放治理。开展“气化焦作”建设，大力推广使用天然气等清洁能源，完成10吨/时及以下燃煤锅炉拆改。在化工、橡胶等11个行业实施挥发性有机物防治。深入开展大气面源污染防治，严格整治建筑工地、道路交通扬尘。加强黄标车限行管理和淘汰。确保大气污染持续下降，有效遏制雾霾天气。

推进碧水工程。强力推进涉水企业深度治理，排查取缔小型制革、造纸等污染严重企业。加快城镇污水处理设施建设，重点推进蟒河孟州段、蒋沟河武陟段等区域水环境综合整治。

开展乡村清洁工程。突出抓好农村人居环境综合整治、生态乡镇生态村创建、畜禽饲养污染治理、重点行业企业清洁生产审核等工作。继续抓好秸秆禁烧，促进秸秆全量化综合利用。

加强生态体系建设。强化废弃矿山地质环境治理，加快南太行山区生态体系、黄（沁）河生态涵养带、南水北调生态产业带及城乡生态廊道网络建设，完成造林任务10.4万亩，确保国家森林城市创建成功。积极申报海绵城市试点，加快构建城市水系，实施人民路水生态、南水北调防洪处理等水系建设工程，打造水通、水清、水美的水生态文明城市。

强化节能减排。加快淘汰落后产能，对符合国家要求的积极申报，进行产能置换。创新有偿使用、预算管理等机制，完善用能权、用水权、排污权、碳排放权初始分配制度及交易市场。

（八）着力增进人民福祉。民之所望，施政所向。集中财力实施一批事关人民群众切身利益的民生工程，织密社会保障安全网，兜住兜牢民生底线，提高公共服务共建能力和共享水平。

持续扩大就业。深入推进统筹城乡就业创业试点市和创业型城市创建，大力推进全民技能振兴工程和职教攻坚工程，继续实施十万个就业岗位推荐、十万人次技能培训、万人创业等专项行动计划，加快创业孵化园区建设，全面落实各项援企稳岗措施，密切关注生产经营困难企业用工动态，新增农村转移劳动力就业2万人，发放创业担保贷款4亿元。

推进脱贫攻坚。大力实施精准扶贫、精准脱贫，以实施太行山区、黄河滩区群众脱贫工程为重点，按照“转、扶、搬、保、救”五条途径，整合扶贫资源，因人因地施策，提高扶贫实效。

强化社会保障。切实保障好优抚对象、低保对象、受灾群众、弱势群体的基本生活。探索实施困难群众大病补充医疗保险，鼓励商业保险机构参与医保经办，完善重大疾病保障体系。继续扩大社会保险覆盖面，推动城镇医疗保险、新农合保障水平不断提高。扶持养老服务业发展，引导社会力量投资兴办养老机构，加快推进市社会福利院和老年公寓楼建设，积极争取省级养老健康产业园区（基地）项目。

发展社会事业。全面提升教育质量，优化义务教育资源配置，大力发展学前教育、职业教育和民办教育，推进韩公教育园区、映湖路小学、职教园区二期等一批项目建设，规划建设市区东部高中，提高教育信息化水平，提升高等教育办学层次。推进健康焦作建设，深化医疗卫生体制改革，全面推进城市公立医院综合改革，完善基层医疗服务模式，推进家庭责任医生制度和签约服务，鼓励社会力量兴办健康服务业。落实全面两孩政策，改革完善计划生育服务管理。强化食品药品监管，积极推进市食品药品检验检测中心项目建设。大力发展群众体育，提升竞技体育水平，办好市“十三运”等重大体育活动。加强基层综合性文化服务中心和百姓文化超市建设，继续实施一批文化惠民工程。认真做好太极拳申遗工作。

维护社会和谐稳定。加快平安焦作建设，创新立体化社会治安防控体系，依法惩治违法犯罪活动。健全完善安全生产责任体系，强化安全生产监管。严密防范、严厉打击非法集资，深入整治拖欠农民工工资行为。全面推行依法逐级走访，加大信访案件化解力度，确保社会安定有序。

同时，着力推动军民融合深度发展，巩固拓展双拥共建活动成果，积极支持驻焦部队建设和改革。进一步做好民族宗教工作。支持工会、共青团、妇联等人民团体发挥桥梁纽带作用。做好外侨、对台、国资、统计调查、审计、人防、地震、档案、史志、红十字、残疾人事业等工作。

四、切实加强政府自身建设

面对艰巨繁重的发展任务，必须以建设法治政府、效能政府、服务政府、廉洁政府为重点，加快转变政府职能，持续改进工作作风，全面提升政府的执行力和公信力，真正做到为人民服务、受人民监督、让人民满意。

建设法治政府。自觉尊法学法守法用法，坚持依法行政，坚决做到法定职责必须为、法无授权不可为。健全依法科学民主决策机制，严格执行重大行政决策法定程序。加强执法监督，确保规范公正文明执法。启动“七五”普法。严格执行人大及其常委会决议决定，自觉接受人大法律监督、工作监督和政协民主监督，认真办理人大议案、建议和政协提案，主动接受舆论监督和社会监督，广泛听取各方面意见建议。

建设效能政府。注重以专业思维、专业方法、专题研究解决问题，注重理顺运行机制破除矛盾障碍，注重优化改进工作流程提高质量和效能。坚决纠正不作为、乱作为，坚决克服懒政、怠政，坚决惩处失职、渎职。完善服务承诺制、限时办结制、首问负责制、效能考评制等制度，推进各项工作规范化、制度化、科学化。

建设服务政府。牢固树立宗旨意识、公仆意识、职责意识，始终把人民放在心中最高位置，坚持深入基层、密切联系群众，多做暖人心、稳人心、得人心的实事。进一步健全为企业、为群众、为基层服务的各项制度，完善行政服务中心、网上办事大厅、为民服务热线等平台，重信践诺，说到做到，全面提升服务水平。

建设廉洁政府。按照干部清正、政府清廉、政治清明的要求，落实“两个责任”，坚持“一岗双责”，持续抓好惩治和预防腐败体系建设。全面推进政务公开，实现决策、执行、结果公开透明。强化财政支出、工程建设、公共资源交易等重点领域和重要环节的监管，切实扎牢制度笼子。完善厉行节约长效机制，严控“三公”经费支出，以清正廉洁的良好形象促进发展、取信于民。

各位代表！

新蓝图承载新希望，新奋斗开创新未来！让我们紧密团结在以习近平同志为总书记的党中央周围，在省委、省政府和市委的坚强领导下，众志成城、砥砺奋进，为全面加快经济转型示范市和美丽焦作建设而努力奋斗！
